# Advancements in APOE and dementia research: Highlights from the 2023 AAIC Advancements: APOE conference

**DOI:** 10.1002/alz.13877

**Published:** 2024-07-19

**Authors:** Courtney M. Kloske, Michael E. Belloy, Elizabeth E. Blue, Gregory R. Bowman, Maria C. Carrillo, Xiaoying Chen, Ornit Chiba‐Falek, Albert A. Davis, Gilbert Di Paolo, Francesca Garretti, David Gate, Lesley R. Golden, Jay W. Heinecke, Joachim Herz, Yadong Huang, Costantino Iadecola, Lance A. Johnson, Takahisa Kanekiyo, Celeste M. Karch, Anastasia Khvorova, Sascha J. Koppes‐den Hertog, Bruce T. Lamb, Paige E. Lawler, Yann Le Guen, Alexandra Litvinchuk, Chia‐Chen Liu, Simin Mahinrad, Edoardo Marcora, Claudia Marino, Danny M. Michaelson, Justin J. Miller, Josh M. Morganti, Priyanka S. Narayan, Michel S. Naslavsky, Marlies Oosthoek, Kapil V. Ramachandran, Abhirami Ramakrishnan, Ana‐Caroline Raulin, Aiko Robert, Rasha N. M. Saleh, Claire Sexton, Nilomi Shah, Francis Shue, Isabel J. Sible, Andrea Soranno, Michael R. Strickland, Julia TCW, Manon Thierry, Li‐Huei Tsai, Ryan A. Tuckey, Jason D. Ulrich, Rik van der Kant, Na Wang, Cheryl L. Wellington, Stacie C. Weninger, Hussein N. Yassine, Na Zhao, Guojun Bu, Alison M. Goate, David M. Holtzman

**Affiliations:** ^1^ Alzheimer's Association Chicago Illinois USA; ^2^ Department of Neurology and Neurological Sciences Stanford University, Stanford Palo Alto California USA; ^3^ NeuroGenomics and Informatics Center Washington University School of Medicine St. Louis Missouri USA; ^4^ Department of Neurology Washington University School of Medicine, St. Louis, Missouri St. Louis Missouri USA; ^5^ Division of Medical Genetics Department of Medicine University of Washington Seattle Washington USA; ^6^ Institute for Public Health Genetics University of Washington Seattle Washington USA; ^7^ Departments of Biochemistry & Biophysics and Bioengineering University of Pennsylvania Philadelphia Pennsylvania USA; ^8^ Department of Neurology Hope Center for Neurological Disorders Knight Alzheimer's Disease Research Center Washington University School of Medicine St. Louis Missouri USA; ^9^ Division of Translational Brain Sciences Department of Neurology Duke University School of Medicine Durham North Carolina USA; ^10^ Department of Neurology Hope Center for Neurological Disorders Washington University School of Medicine St. Louis Missouri USA; ^11^ Denali Therapeutics Inc. San Francisco California USA; ^12^ Ronald M. Loeb Center for Alzheimer's Disease New York New York USA; ^13^ Department of Genetics & Genomic Sciences Icahn School of Medicine at Mount Sinai New York New York USA; ^14^ The Ken & Ruth Davee Department of Neurology Northwestern University Feinberg School of Medicine Chicago Illinois USA; ^15^ Department of Physiology University of Kentucky Lexington Kentucky USA; ^16^ Sanders‐Brown Center on Aging University of Kentucky Lexington Kentucky USA; ^17^ Department of Medicine University of Washington, UV Medicine Seattle Washington USA; ^18^ Center for Translational Neurodegeneration Research University of Texas Southwestern Medical Center Dallas Texas USA; ^19^ Gladstone Institute of Neurological Disease Gladstone Institutes San Francisco California USA; ^20^ Department of Neurology University of California San Francisco California USA; ^21^ Feil Family Brain and Mind Research Institute Weill Cornell Medicine New York New York USA; ^22^ Department of Neuroscience Mayo Clinic Jacksonville Jacksonville Florida USA; ^23^ Department of Psychiatry Washington University in St Louis St. Louis Missouri USA; ^24^ RNA Therapeutic Institute UMass Chan Medical School Worcester Massachusetts USA; ^25^ Department of Functional Genomics Center for Neurogenomics and Cognitive Research (CNCR) VU University Amsterdam Amsterdam USA; ^26^ Alzheimer Center Amsterdam Department of Neurology Amsterdam Neuroscience, Amsterdam University Medical Center Amsterdam USA; ^27^ Stark Neurosciences Research Institute Indiana University School of Medicine Indianapolis Indiana USA; ^28^ The Tracy Family SILQ Center Washington University School of Medicine Indianapolis Indiana USA; ^29^ Department of Neurology and Neurological Sciences Stanford University Palo Alto California USA; ^30^ Institut du Cerveau–Paris Brain Institute–ICM Paris France; ^31^ Department of Genetics and Genomic Sciences Nash Family Department of Neuroscience Icahn Genomics Institute; Icahn School of Medicine at Mount Sinai New York New York USA; ^32^ Schepens Eye Research Institute of Mass Eye and Ear and Department of Ophthalmology at Harvard Medical School Boston Massachusetts USA; ^33^ Sagol School of Neuroscience Tel Aviv University Tel Aviv Israel; ^34^ Department of Biochemistry and Molecular Biophysics Washington University School of Medicine St. Louis Missouri USA; ^35^ Department of Neuroscience University of Kentucky Lexington Kentucky USA; ^36^ Genetics and Biochemistry Branch National Institute of Diabetes and Digestive and Kidney Diseases National Institute of Neurological Disorders and Stroke Center for Alzheimer's and Related Dementias (CARD) National Institutes of Health Maryland USA; ^37^ Human Genome and Stem‐cell Research Center Biosciences Institute University of São Paulo Rua do Matao São Paulo Brazil; ^38^ Hospital Israelita Albert Einstein Avenida Albert Einstein São Paulo Brazil; ^39^ Neurochemistry Laboratory Department of Laboratory Medicine Vrije Universiteit Amsterdam, Amsterdam UMC Amsterdam Netherlands; ^40^ Taub Institute for Research on Alzheimer's Disease and the Aging Brain Columbia University Vagelos College of Physicians and Surgeons New York New York USA; ^41^ Department of Neurology Columbia University Irving Medical Center New York New York USA; ^42^ Department of Neuroscience Columbia University Vagelos College of Physicians and Surgeons New York USA; ^43^ Mayo Clinic Florida Jacksonville Florida USA; ^44^ Norwich Medical School University of East Anglia, UK Clinical and Chemical Pathology Norfolk UK; ^45^ Faculty of Medicine Alexandria University Alexandria Governorate Egypt; ^46^ Lexeo Therapeutics New York New York USA; ^47^ University of Southern California Los Angeles California USA; ^48^ Washington University in Saint Louis, St. Louis, Missouri, USA St. Louis Missouri USA; ^49^ Department of Pharmacology Physiology & Biophysics Chobanian and Avedisian School of Medicine Boston University Boston Massachusetts USA; ^50^ Bioinformatics Program Faculty of Computing & Data Sciences Boston University Boston Massachusetts USA; ^51^ Center for Cognitive Neurology Department of Neurology New York University Grossman School of Medicine New York New York USA; ^52^ Picower Institute for Learning and Memory Department of Brain and Cognitive Sciences Massachusetts Institute of Technology Cambridge Massachusetts USA; ^53^ Department of Neurology Center for Neurodegeneration and Experimental Therapeutics Medical Scientist Training Program University of Alabama at Birmingham Birmingham Alabama USA; ^54^ Mayo Clinic Rochester Rochester Minnesota USA; ^55^ Djavad Mowafaghian Centre for Brain Health Department of Pathology and Laboratory Medicine International Collaboration on Repair Discoveries School of Biomedical Engineering University of British Columbia Vancouver Canada; ^56^ FBRI Massachusetts USA; ^57^ Department of Neurology Keck School of Medicine University of Southern California Los Angeles California USA; ^58^ Division of Life Science Hong Kong University of Science and Technology Clear Water Bay Kowloon Hong Kong; ^59^ Department of Genetics & Genomic Sciences Ronald M. Loeb Center for Alzheimer's disease Icahn School of Medicine at Mount Sinai New York New York USA

**Keywords:** Alzheimer's disease, APOE, apolipoprotein E, conference proceedings, dementia, lipids, microglia, neuroinflammation, risk factor, therapeutics, vasculature

## Abstract

**INTRODUCTION:**

The apolipoprotein E gene (*APOE*) is an established central player in the pathogenesis of Alzheimer's disease (AD), with distinct apoE isoforms exerting diverse effects. apoE influences not only amyloid‐beta and tau pathologies but also lipid and energy metabolism, neuroinflammation, cerebral vascular health, and sex‐dependent disease manifestations. Furthermore, ancestral background may significantly impact the link between *APOE* and AD, underscoring the need for more inclusive research.

**METHODS:**

In 2023, the Alzheimer's Association convened multidisciplinary researchers at the “AAIC Advancements: APOE” conference to discuss various topics, including apoE isoforms and their roles in AD pathogenesis, progress in apoE‐targeted therapeutic strategies, updates on disease models and interventions that modulate apoE expression and function.

**RESULTS:**

This manuscript presents highlights from the conference and provides an overview of opportunities for further research in the field.

**DISCUSSION:**

Understanding apoE's multifaceted roles in AD pathogenesis will help develop targeted interventions for AD and advance the field of AD precision medicine.

**Highlights:**

- APOE is a central player in the pathogenesis of Alzheimer's disease.
- APOE exerts a numerous effects throughout the brain on amyloid‐beta, tau, and other pathways.
- The AAIC Advancements: APOE conference encouraged discussions and collaborations on understanding the role of APOE.

## 1 INTRODUCTION

In 1973, researchers identified an arginine‐rich protein in the very low‐density lipoprotein (VLDL) of patients with a rare lipid metabolism disorder called familial hypercholesterolemia type III.^1^ This protein was further characterized and named apolipoprotein E, or apoE,^2^ which is encoded by the apolipoprotein E gene (*APOE*) and has three main alleles: *ε2, ε3*, and *ε4* that translate to three isoforms: apoE2, apoE3, and apoE4, respectively.^3^ The apoE protein was later found to play a role in lipid metabolism, cardiovascular disease (CVD), and Alzheimer's disease (AD).^4^, ^5^, ^6^, ^7^, ^8^

Over the past decades, apoE has attracted greater attention in AD research. The *APOE ε2* allele has been determined to be protective against AD, while the *ε4* allele was found to be associated with increased AD risk.^7^, ^8^, ^9^ Research has also found an important correlation between the frequency of APOE variants across different populations and their associated risk for AD pathology.^10^ This landscape of findings has opened an important avenue for a more precise mechanistic understanding of AD etiology and pointed toward numerous potential treatment targets. However, it is important to note that most research in the area of apoE and AD has focused on non‐Hispanic White populations, leading to a lack of diversity in the field and undermining the breadth and impact of apoE and AD research on diverse populations.

The Alzheimer's Association convened the Alzheimer's Association International Conference (AAIC) Advancements: APOE conference on March 6‐7, 2023, to foster new collaborations and develop novel research directions with the potential to break down multidisciplinary barriers and drive transformative neuroscience research. This manuscript provides an overview of the discussions from this conference while highlighting knowledge gaps in the field that need to be addressed by future research.

## 2 THE APOE GENE AND NEURODEGENERATIVE DISEASE RISK

The *APOE ε4* has been identified as the most significant genetic risk factor associated with AD^11^ and other neurodegenerative diseases, including frontotemporal lobar dementia (FTLD), Lewy body dementia (LBD), and other amyloid‐beta (Aβ) and tau pathologies.^12^ While *APOE ε2* is established as protective against AD, *APOE ε4* is generally associated with greater AD risk if using *APOE* ε3 as a reference allele. In addition, *APOE* ε4 is associated with decreased age of AD onset and promotion of Aβ and tau pathology, inflammation, and neurodegeneration.^13^ Pathways associated with the risk of AD and other dementia, such as lipid metabolism, cardiovascular and cerebrovascular diseases, altered efferocytosis, inflammation, trafficking, and integrated stress response, are also associated with *APOE*.^13^, ^14^, ^15^, ^16^, ^17^, ^18^ By interrogating the relationship between *APOE* and various disease states and pathways, researchers have learned that *APOE* can influence AD risk through a variety of pathways.

Several rare *APOE* variants have also been identified. For example, the rare *APOE*‐Ch (R136S) mutation, located within the overlapping N‐terminal heparan sulfate proteoglycan (HSPG) and receptor binding domain of apoE,^4^, ^19^ was identified in a Colombian kindred with a dominant PSEN1 E280A mutation.^20^ While *PSEN1* mutation carriers often exhibit significant Aβ and tau and burden, hippocampal atrophy, and brain hypometabolism,^21^, ^22^ *APOE*‐Ch carriers with *PSEN1* mutation have been shown to be protective against AD, with one homozygous individual exhibiting minimal tau pathology, hippocampal atrophy, and hypometabolism despite the presence of a *PSEN1* mutation.^20^, ^21^ Studies using the Alzheimer's Disease Sequencing Project (ADSP) data have identified several other rare *APOE* variants associated with AD, including the R145C commonly found in the African American AD population.^23^ The R145C variant causes a heterozygotic effect, associated with an increased risk of AD and reduced age of onset only in the presence of an ε4 allele.^24^ Insights from the conference highlight *APOE* variants identified in populations of European ancestry including L28P, V236E (also known as the Jacksonville variant), and R251G. Studies have shown that the V236E and R251G variants are associated with decreased AD risk, whereas the L28P variant has not been associated with AD risk.^23^, ^24^

**RESEARCH IN CONTEXT**

1. **Systematic review**: The role of apolipoprotein E (APOE) in neurodegenerative diseases, including Alzheimer's and other dementia, is an active and growing area of research. The authors of this manuscript report updates and advances in research presented at the 2023 AAIC Advancements: APOE Conference, held in March of 2023.
2. **Interpretation**: There have been strides in research identifying the role of APOE in dementia research. This manuscript highlights the research presented at the 2023 AAIC Advancements APOE Conference including the role of apoE isoforms and their roles in AD pathogenesis, progress in apoE‐targeted therapeutic strategies, and updates on disease models and interventions that modulate apoE expression and function. This manuscript also highlights apoE influences not only amyloid‐beta and tau pathologies but also lipid and energy metabolism, neuroinflammation, cerebral vascular health, and sex‐dependent disease manifestations. Furthermore, ancestral background may significantly impact the link between APOE and AD, underscoring the need for more inclusive research.
3. **Future directions**: Understanding apoE's multifaceted role in AD pathogenesis will help develop targeted interventions for AD and advance the field of AD precision medicine.

## 3 RACIAL AND ETHNIC DIFFERENCES IN APOE AND AD RISK

Although significant progress has been made in understanding the link between APOE and AD, most studies have focused on non‐Hispanic White populations and do not adequately represent human diversity.^25^ The *AAIC advancements: APOE conference* emphasized the need to explore and address population‐level distinctions in APOE and AD, particularly through research involving diverse populations. For example, several presentations highlighted that the frequency of *APOE* ε4 varies substantially across different populations, with central Africa and northern Europe displaying the highest *APOE* ε4 frequencies.^25^ Among European, African American, Hispanic, and Japanese populations, *APOE* ε4/ε4 homozygotes are most common in Japanese populations.^26^

The three most common *APOE* alleles appear within the general global population at varying frequencies. The *APOE* ε3 allele is the most common across all human populations, with frequencies ranging from 85% in Asia to 69% in Africa. The *APOE* ε4 allele is enriched in indigenous populations of Central Africa, Oceania, and Australia, with frequencies ranging from 26% to 40%, while the Mediterranean area or south China has a low frequency of <10%. The *APOE* ε2 allele is the least common, with a worldwide frequency of about 7% and no apparent geographical trend.^13^ Overall, accumulating evidence suggests that the frequencies of the major *APOE* alleles differ dramatically across geographical, racial, and ethnic groups, the genetic‐environmental interplay heterogeneously impacts disease risk within those populations,^27^, ^28^, ^29^ and *APOE*‐related risk of AD may be sex‐dependent.^29^, ^30^

Furthermore, research has shown that the impact of *APOE* genotype on AD risk varies across populations with diverse ancestral backgrounds. For example, in Caribbean Hispanic individuals, African‐derived ancestry of *APOE* genotype is associated with a lower risk of AD compared to individuals with European‐derived *APOE* genotype.^31^ These results are consistent with studies in African American and Puerto Rican populations.^32^ Therefore, a commitment to the diversification of *APOE* and AD research is needed to expand the understanding of the role of *APOE* in AD for diverse populations affected by the disease.

## 4 STRUCTURE OF THE APOE PROTEIN AND ITS BIOCHEMICAL PROPERTIES

apoE is the most abundant apolipoprotein in the brain^33^ and, depending on the isoform, can either contribute to AD pathologies or protect against them. Initially studied in hyperlipidemia, *APOE* encodes a 317‐amino‐acid protein, apoE, that includes an 18‐residue signal peptide. Cleavage of this signal peptide, followed by glycosylation at one of the several glycosylation sites within the sequence, results in the mature apoE protein, 299 amino acids in length and approximately 34 kDa. This protein's tertiary structure comprises two independently folded domains separated by a hinge region. The N‐terminal domain includes the receptor binding domain and one of two heparin‐binding regions. The C‐terminal domain includes the lipid‐binding domain and the other heparin‐binding region. The work presented at the conference and through this section suggests these domains enable the spectrum of interactions and functions that make apoE a critical component of various neurodegenerative diseases.^34^

### 4.1 Conformational heterogeneity in apoE protein

While apoE plays a significant role in AD pathology, the structural determinants of apoE that contribute to this pathogenicity remain unclear. In vivo, apoE is secreted primarily by astrocytes in discoidal HDL‐like lipoproteins.^35^, ^36^, ^37^ Under disease conditions, microglia upregulate apoE expression.^38^, ^39^ Research suggests that apoE can exist in various conformational states in lipid‐free and lipid‐bound forms, which may have important implications for its biological functions.^40^ apoE undergoes extensive conformational changes upon binding to lipids. The four‐helix bundle in nonlipidated apoE unfolds, and the hydrophobic portions of the amphipathic α‐helix associate with lipid.^41^, ^42^ This conformational change is critical to apoE's function as nonlipidated apoE is unable to bind low‐density lipoprotein receptor (LDLR).^43^, ^44^

The structural changes accompanying lipidation are poorly understood. The monomeric form of the nonlipidated apoE has been proposed as the competent form for lipid binding.^45^ However, structural characterization has been elusive because of the strong propensity of apoE for oligomerization, with dimers already forming at a nanomolar concentration.^45^, ^46^, ^47^ As a result, the structural features of nonlipidated apoE have been determined only for N‐terminal domain fragments that lack the C‐terminal domain^46^, ^47^ and for a “monomeric” mutant of apoE ε3 that contains several mutations in the C‐terminal domain.^48^ To close this knowledge gap and overcome these experimental challenges, single‐molecule Förster resonance energy transfer (smFRET) was implemented to isolate apoE4 monomers and measure distances between fluorescently labeled positions of apoE4.^40^ smFRET revealed three major conformational ensembles of apoE, which differ largely for the conformations of the C‐terminal domain with respect to the closed, open, and extended N‐terminal domains. Furthermore, smFRET experiments using DMPC liposomes to analyze lipidated apoE4 revealed both compact and expanded conformations.^40^

### 4.2 Biochemical and structural changes in rare APOE variants

Recombinant forms of apoE ε3 variants—apoE3‐Christchurch (apoE3‐Ch) and *APOEapoE3*‐R145C, but not apoE3‐Jacksonville (apoE3‐Jac)—show reduced heparin‐binding affinity and mixed LDLR binding affinity.^20^, ^49^, ^50^

Molecular dynamics simulations allow for comparing conformational and structural characteristics of common and rare apoE isoforms when in a closed conformation. Hydrogen bond occupancy analyses between common apoE isoforms, apoE ε3, apoE ε2, and apoE ε4, support prior findings that apoE ε2 has altered hydrogen bond occupancy between residues in the receptor binding domain.^51^ In addition, all apoE isoforms show similar conformational flexibility measured via root mean square fluctuation; however, the C112R substitution present in apoE ε4 may increase conformational motion in specific functional domains of apoE ε4 compared to apoE ε3 and apoE ε2. While the rare variant apoE4‐R251G has the same C112R substitution, it shows a distinct pattern of conformational motion in these same regions compared to apoE ε4.

### 4.3 Post‐translational modifications in apoE

Post‐translational modifications (PTMs) are chemical transformations that proteins undergo after translation, resulting in a functional alteration for the protein. Many proteins associated with AD undergo PTMs, including apoE, APP, tau, and Aβ. In most cases, PTMs are imperative to the proper functioning of a protein. Such PTMs can directly affect apoE structure and function. However, some PTMs may impede protein function and result in downstream consequences. One particularly critical PTM in AD is glycosylation, a process required to produce mature apoE.^52^ Immunoprecipitation and mass spectrometry assessing O‐glycosylated apoE in plasma and cerebrospinal fluid (CSF) samples of older adults showed that CSF‐derived apoE has a higher proportion of total glycosylation than plasma‐derived apoE.^52^ In addition, plasma‐derived apoE O‐glycosylation levels differ significantly by *APOE* genotype and CSF amyloid status, with *APOE* ε4/ε4 carriers showing low apoE O‐glycosylation. These findings presented at the conference suggest that O‐glycosylation of apoE could be a potential CSF or blood‐based biomarker for brain amyloidosis and AD diagnosis.

### 4.4 Role of apoE and ABCA1 in lipid transport and metabolism

In addition to modifications to APOE, APOE also plays an important role in interacting with other proteins. The adenosine triphosphate (ATP)‐binding cassette transporters A1 (ABCA1) and apoE are both critical players in lipid metabolism, particularly in the context of lipid transport in the central nervous system (CNS).^53^ In high‐density lipoprotein (HDL) particles, apoA1 binds ABCA1, leading to the translocation of cholesterol across the phospholipid bilayer membrane, thereby regulating cholesterol efflux. ABAC1 is a critical element to consider in AD studies because of its potential relevance to apoE metabolism and supporting apoE protein levels in the CNS. Given that research suggests a link between ABCA1, apoE levels in the CNS, and AD pathology, proper ABCA1 functioning is important to understanding apoE metabolism and AD progression. While the structure of APOE is necessary to consider, the additional functions of APOE were also discussed at the AAIC Advancements conference.

## 5 *APOE*, MICROGLIAL FUNCTION, IMMUNOMODULATION, AND CHANGES TO GLIAL LIPID METABOLISM IN AD

The mechanisms underlying the pathogenic effects of apoE in AD are complex and involve multiple pathways.^54^ apoE is involved in several potentially pathologic processes that may lead to the development of dementia. For example, apoE isoforms are linked to differential levels of Aβ‐accumulation in the brain, with apoE ε4 associated with the highest accumulation levels and apoE ε2 associated with the lowest.^55^ In addition, apoE plays a role in blood‐brain barrier (BBB) integrity and induction of microglia‐driven phagocytosis and inflammation. apoE ε4 is associated with BBB dysfunction, increased phagocytosis, and proinflammatory activity.^56^, ^57^, ^58^ A growing number of studies suggest that apoE mediates the induction of disease‐associated microglia (DAM).^38^ apoE has also been implicated in several metabolic abnormalities, including abnormal glucose metabolism, altered lipidome and metabolome, mitochondrial dysfunction, and decreased oxygen consumption, all of which are involved in the pathology of dementias.^59^, ^60^, ^61^, ^62^, ^63^ The AAIC Advancements: APOE conference highlighted new data to help delineate the mechanisms underlying apoE's contribution to AD‐associated pathologies.

Evidence suggests that inflammation and immunomodulation pathways play a role in the pathogenesis of AD. Pathways that rely on or are modulated by specific AD risk factors, including triggering receptors expressed on myeloid cells 2 (TREM2) and specific cell types, such as glial cells, have been implicated in AD. The combination of these risk factors, cellular changes, and individual *APOE* genotypes results in the heterogeneous immunophenotypes observed in AD.^64^

### 5.1 apoE in microglial functions and AD

Research from mouse models of AD pathology suggests that while the microglial response to Aβ is generally protective against axonal injury, microglial responses to tau pathology promote neurodegeneration. In Aβ and tau pathology, microglia exhibit TERM2 and apoE‐dependent upregulation of genes, colloquially referred to as DAM. apoE isoforms may differentially impact microglial activation in a cell‐autonomous manner.^64^ One study presented at the conference suggested microglial apoE ε3 expression improves cognitive behavior, synaptic function, and microglial responses to injury in a tamoxifen‐inducible transgenic animal model, iE3/Cx3cr1‐Cre^ER^ mice, while microglial from iE4/Cx3cr1‐Cre^ER^ elicits minimal or an opposite effect. Furthermore, microglial apoE ε3 expression increases plaque‐associated microgliosis and reduces Aβ deposition and associated neuronal toxicity, whereas microglial apoE ε4 expression either compromises or has no effects on these outcomes by impairing lipid metabolism.^65^

In addition, research has shown that TREM2 can interact with apoE and its isoforms to induce microgliosis.^66^ Research presented at the conference showed that TREM2‐independent microgliosis promotes tau‐mediated neurodegeneration in the presence of APOE4, increasing neutral lipid accumulation in microglial phagolysosomes.^67^ TREM2 deletion does not counteract the detrimental effect of apoE ε4 on tau‐mediated neurodegeneration and synaptic loss, nor does it lower tau pathology or attenuate microglial lysosomal burden. Homeostatic microglial markers are preserved despite neurodegeneration in Tau‐APOEε4 (TE4)‐TREM2 knockout (KO) mice. However, some reactive microglial markers, such as Clec7a, are reduced by TREM2 KO in TE4 mice. TE4 and TE4‐TREM2 KO mice display increased lysosomal enzymes; thus, TREM2 KO increases phagolysosome volume in TE4 mice.^67^

#### 5.1.1 apoE‐modulated microglial response to myelin damage

Many of the identified 75 genomic loci that impact the risk of AD regulate lipid metabolism.^11^ Recently, it has been shown that apoE ε4 is associated with gene expression changes in cholesterol‐ and lipid‐related pathways across all human brain cell types.^10^, ^68^ This dysregulated cholesterol activity is associated with reduced myelination in apoE ε4 carriers. Solubilizing cholesterol relieves the cholesterol burden, and treatment with cyclodextrin increases myelination in apoE ε4 culture and restores axonal myelination in apoE ε4 KI mice. Notably, cyclodextrin‐treated mice exhibit greater interhemispheric fast gamma phase‐locking, associated with improved cognition.^68^

In response to myelin damage, microglia clear myelin debris, secrete regenerative factors, and modulate the extracellular matrix. Microglial response to myelin damage is a common pathological feature in neurodegenerative disorders. To determine how apoE isoforms affect microglial responses to myelin damage, a recent study by Wang et al. used the apoE‐targeted replacement (TR) mouse models fed with normal diet or cuprizone to induce demyelination. Significant isoform‐dependent differences in microglial activation and function were observed. apoe ε4‐TR mice had more myelin debris accumulation than apoe ε2‐TR mice upon cuprizone treatment. The microglia in apoe ε2‐TR mice proliferated more and demonstrated hyperactivity compared to apoe ε4‐TRmice. Genes related to the immune response, inflammatory signaling, and lipid metabolism were upregulated in apoe ε2‐TR mice and downregulated in apoe ε4‐TRmice. Moreover, apoe ε4 microglia had a reduced ability to clear myelin debris due to a weaker phagocytosis ability and enhanced lipid droplet accumulation than apoe ε2 microglia.^69^ Thus, apoE isoforms may differentially regulate microglia activation and lipid metabolism in the context of myelin damage.

### 5.2 apoE, immunomodulation, and immunometabolism in AD

A study presented at the conference demonstrated that mice with tauopathy, but not Aβ, developed a unique innate and adaptive immune response and that the depletion of microglia or T‐cells or inhibition of interferon‐gamma (IFN‐γ) signaling can significantly ameliorate brain atrophy and improve cognitive behavior. Consistent with the finding that apoE ε4 exacerbates tau‐mediated neurodegeneration, T cell infiltration increases in mice with apoE ε4 but did not present in the tau mice lacking apoE. These data suggest apoE is important in immunomodulation and may link innate and adaptive immunity.^70^, ^71^

Spatial transcriptomics in 5XFAD mice expressing apoE ε4 revealed a unique cortical transcriptomic signature characterized by increased expression of DAM/MGnD microglia genes and biomarkers related to microglial activation, lipid metabolism, complement activation, and synapse pruning.^72^ Furthermore, in the presence of both advanced age and Aβ overexpression, apoE ε4 exacerbated expression of plaque‐induced genes (PIGs), decreased expression of myelin and oligodendrocyte genes (OLIGs), and was associated with transcriptomic signatures related to microglial activation and lipid metabolism.^73^ Finally, when analyzing the brain in a spot‐by‐spot manner, increases in local plaque load were highly correlated with changes in genes related to lipid metabolism in apoE ε4 but not apoE ε3, expressing 5XFAD mice. These data suggest that apoE ε4 negatively impacts microglial function and alters lipid metabolism, which in concert may lead to the dysregulated immunometabolism characteristic of AD.^72^

### 5.3 apoE and glial lipid metabolism

Lipids play a major role in the processes that are associated with AD pathogenesis, including synaptic plasticity, inflammation, and oxidative stress. Glial lipid metabolism is one of the several pathways implicated in AD pathology.^74^ Researchers have interrogated the relationships between AD pathologies and lipid metabolism, such as the association of specific apoE isoforms and calcium‐dependent phospholipase A2 (cPLA2) activation.^75^ For example, apoE ε4 increases calcium‐dependent cPLA2 activation, leading to accentuated eicosanoid lipid metabolism and neuroinflammation. Compared to individuals with the apoE ε3/ε3 genotype, astrocytes from apoE ε4 carriers exhibit increased cPLA2 expression, particularly surrounding Aβ plaques. Notably, cPLA2 reduction ameliorates cognitive deficits in an AD mouse model and resolves chronic neuroinflammation from low docosahexaenoic acid DHA diets in apoE ε4‐TR mice and increases brain DHA and eicosapentaenoic acid (EPA) levels.^76^ cPLA2 inhibition also reverses acute LPS‐induced inflammation. No cPLA2 inhibitor has progressed to human studies; future research will involve examining small molecules that inhibit cPLA2 for mitigating neuroinflammation.

Another major piece of lipid metabolism involves cholesterol.^77^, ^78^ Cholesterol metabolism is dysregulated in both *post mortem* AD brains and animal AD models, leading to cholesterol ester accumulation in the brain and glial cells in a manner consistent with defects in cholesterol efflux. apoE ε4 microglia display cholesterol dysregulation consistent with a cholesterol ester storage disorder.^79^, ^80^, ^81^, ^82^, ^83^ A recent study presented at the conference suggests the potential mechanism underlying dysregulated cholesterol metabolism in apoE ε4/ε4 astrocytes. A change in intracellular cholesterol distribution causes cells to upregulate de novo cholesterol synthesis and increase cell surface lipid receptors to facilitate cholesterol uptake.^84^ Simultaneously, reduced phagocytic capacity, apoE levels, and lipid transporter levels lead to decreased cholesterol secretion and efflux. apoE ε4/ε4 astrocytes exhibit an overall decrease in genes associated with the autophagy pathway and network, especially lysosomal acidification, membrane, biogenesis, and hydrolases. Compared to apoE ε3/ε3 astrocytes, apoE ε4/ε4 astrocytes exhibit more LDL binding but reduced uptake of lipids, exposing lipids on cell surfaces and leading to reduction of adhesion to the actin cytoskeleton by apoE ε4/ε4 astrocytes. Furthermore, enriched matrisome pathways associated with upregulated chemotaxis, glial activation, and lipid biosynthesis originate from astrocytes in the presence of neurons in the AD brain.^84^

Another way to interrogate the relationship between AD and lipid metabolism is through unbiased lipidomics analysis of induced pluripotent stem cells (iPSC)‐derived brain cells. Lipidomics data demonstrates that *APOE* genotypes uniquely affect lipid metabolism in iPSC‐microglia, neurons, and astrocytes. Compared to apoE ε3, apoE ε4 microglia and astrocytes display increased triglycerides^85^ and cholesterol ester levels.^86^, ^87^ Proteomic analyses showed an upregulation of mitochondrial pathways in apoE ε4 and apoE KO astrocytes, whereas the same pathways are downregulated in the apoE ε4 and apoE KO microglia. Additionally, apoE ε4 glia increased cholesterol synthesis and altered immune signaling, indicating tight coupling between intracellular cholesterol distribution and immune activity of glia.^86^, ^87^

Using a novel conditional mouse model, researchers have identified that a “midlife midlife switch” involving a full‐body transition from the expression of apoE ε4 to apoE ε2 impacts the cerebral transcriptome and lipidome. Single‐cell RNA sequencing identified astrocytes, oligodendrocytes, endothelial cells, and microglia as the cell types most impacted by the apoE ε4 to apoE ε2 “switch.” Each of these cell types has differentially expressed genes that have been associated with AD‐related pathways, particularly pathways involved with lipid and carbohydrate metabolism. Lipidomic analyses further showed that several glycerophospholipid species, including phosphatidylcholines, were impacted by the midlife apoE ε4:apoE ε2 allele switch, which is consistent with previous *post mortem* human AD studies. These findings suggest that apoE ε4's transcriptional and lipidomic signatures are not set in stone during development or early life. Rather, these signatures appear acutely malleable within the specific parameters tested here when mice are switched at a “mid‐lifemidlife’” (6 months) and assessed 1 month post apoE ε4:apoE ε2 switch.^88^

Using unbiased lipidomics coupled with immunostaining, a recent study demonstrated that apoE ε4 promotes cholesterol ester accumulation in the endolysosomal compartment of microglia of aged P301S tauopathy mice when compared to P301S mice expressing apoE ε3 or apoE KO animals.^83^ Increasing cholesterol efflux via LXR agonist diet or by ABCA1 overexpression significantly decreased lipid droplet accumulation in vitro and dramatically reduced tau pathology and associated neurodegeneration, neuroinflammation, and microglial cholesterol ester accumulation in vivo. These data demonstrate that promoting cholesterol efflux could be a beneficial therapeutic approach to reduce tau pathology and apoE ε4‐linked neurodegeneration.

## 6 APOE AND NEUROVASCULAR DYSFUNCTION IN AD

### 6.1 Role of apoE receptors in cerebrovascular integrity

apoE ε4 has been previously shown to impair neurovascular integrity, which may in turn enhance neurodegeneration.^89^ At the conference, research presented highlighted that deleting the major apoE receptor LRP1 affects the cerebrovascular system and cognitive performance in a genotype‐dependent manner. Specifically, impaired spatial memory, excessive perivascular glial activation, reduced cerebrovascular collagen IV, and disrupted BBB integrity were observed in apoE ε4 mice, but not in apoE ε3 mice with smLrp1 removed. This suggests that LRP1 in vascular mural cells maintains cerebrovascular integrity and functions in an apoE genotype‐dependent manner.^90^

One mechanism underlying the link between apoE ε4 and impaired neurovascular integrity could be attributed to BBB leakage. Human AD brains show widespread decreases in tight junction proteins, which are critical for maintaining BBB integrity.^10^ *APOE* ε4 carriers display BBB breakdown in the hippocampus and medial temporal lobe, while noncarriers do not. This BBB breakdown increases with impaired cognition and is independent of CSF Aβ and CSF tau presence.^57^

### 6.2 Role of border‐associated macrophages

Recent findings also suggest that apoE ε4‐induced neurovascular dysfunction may be mediated by border‐associated macrophages, specifically perivascular macrophages (PVM). A recent study presented at the conference showed that apoE ε4, but not apoE ε3, induces reactive oxygen species (ROS) production in PVM in vivo and ex vivo. Conditional deletion of apoE ε4 in PVM rescues neurovascular dysfunction, indicating that PVM cells are both the source and target of apoE ε4 and drive neurovascular dysfunction.^91^

### 6.3 apoE and blood pressure variability in dementia

Blood pressure variability (BPV) has also emerged as a risk factor for dementia, particularly in individuals with apoE ε4. Elevated BPV, independent of mean BP, has been associated with cognitive decline, AD progression, and cerebrovascular disease burden. Interestingly, a recent observational study found that only people with apoE ε4 isoform displayed an association between high BPV and cognitive decline.^92^ The accelerated cognitive decline among apoE ε4 carriers may be due to the “tsunami effect,” in which large fluctuations of BP exacerbates apoE ε4‐associated vulnerability, such as a leaky BBB. Such findings provide opportunities to develop targeted interventions for *APOE* ε4 carriers with high BPV.^92^

## 7 MODELS OF APOE PHENOTYPES

Both animal and iPSC models are important not only for understanding the pathology and biological mechanisms of apoE but also for developing therapeutics. These models provide opportunities for preclinical therapeutic testing once researchers have identified mechanisms for modifying apoE function. Although iPSC lines enable researchers to study molecular mechanisms, the AD research community has primarily used animal models to characterize apoE's broad physiological effects. Recently, researchers have developed humanized mouse models by using knock‐in (KI) techniques to integrate human *APOE* into the mouse genome.^93^

### 7.1 Mouse models

The first KI mouse model of human APOE was developed by researchers at Duke University, which is now distributed through Taconic Biosciences. The Jackson Laboratory (Jax) Model‐AD Consortium also developed KI mouse models of apoE ε3 and apoE ε4 on the C57BL6/J background. Jax is currently developing models of the apoE ε3‐Ch and apoE ε3‐Jac variants, as well as inducible apoE reporters and flips of apoE ε4 to ε3, apoE ε3 to ε4, and apoE ε3 to ε2. The Cure Alzheimer's Fund also has a KI model with floxed alleles that allow for conditional deletion.^93^ Understanding the limitations of each model is important in deciphering the results from studies and furthering the understanding of the field.

Most phenotypes of Jax and Taconic mouse models are reproducible, but some researchers have not observed increases in SERPINA3 as previously described in the Taconic model. In addition, apoE expression levels are typically higher in Jax mice compared to the Taconic mice, which may be due to a leftover neomycin cassette in the Taconic model. Other phenotypic changes are limited by age, as these mouse models do not show behavioral differences until 16 months old. However, Jax has observed sex‐dependent changes in vascular coupling from fluorodeoxyglucose (FDG) positron emission tomography (PET) by 12 months old.

### 7.2 Other animal models

Using rats as an apoE model can potentially facilitate greater characterizations in vascular changes and CSF flow. In addition, the Jax Marmo‐AD program aims to develop an early‐onset AD marmoset model harboring a *PSEN1* mutation and a late‐onset AD marmoset model harboring an *ABCA7* mutation. However, nonhuman primate (NHP) models can limit study sample sizes and increase study timelines due to challenges in producing large, aged cohorts of NHPs.^93^

## 8 THERAPEUTIC STRATEGIES

A range of therapeutic strategies targeting apoE or apoE signaling mechanisms and building on recent technological advances are in various stages of development. These approaches include genetic therapies such as an adeno‐associated virus (AAV), antisense oligonucleotides (ASOs), and RNA interference (RNAi); antibodies; and small‐molecule drugs. Incorporating refined trial designs and biomarker developments may facilitate meaningful mechanistic insight regardless of trial outcomes.^94^

### 8.1 RNAi modulation of apoE expression

Using RNAi to silence disease‐associated genes on demand is a particularly attractive strategy in the context of neurodegeneration. The entrapment of siRNA compounds in the endolysosomal system creates a slowly released intracellular depot of drug that enables multimonth efficacy. In addition, optimization of chemical scaffolds for DNA delivery (i.e., dianophores) enables the selective delivery of siRNAs to the CNS or other target tissues.^95^ The recent development of a novel divalent siRNA dianophore enables potent and sustained modulation of gene expression throughout the CNS of mice as well as larger NHPs, specifically reducing gene expression in the CNS by more than 99% for up to 12 months.^96^

RNAi for apoE ε4 is currently under development as a novel therapeutic approach for treating late‐onset AD (LOAD). Genetic evidence suggests that in the CNS, apoE ε4 promotes neurodegeneration through a toxic gain‐of‐function mechanism^55^, ^77^, ^97^ and that removing apoE completely in a mouse model of tauopathy blocks most tau‐mediated neurodegeneration^98^ and decreasing apoE ε4 in the brain by ∼50% using antisense oligonucleotides also significantly reduces tau‐mediated neurodegeneration.^99^ However, because apoE is critical for systemic cholesterol metabolism, maintaining the systemic function of apoE is essential.^100^, ^101^, ^102^ These findings constrain the development of RNAi to target apoE ε4.

### 8.2 Correcting endolysosomal dysfunction mediated by apoE ε4

Converging lines of research implicate dysfunction of the endolysosomal system in aging and AD and suggest that apoE ε4 exacerbates these deficits.^14^, ^15^, ^103^, ^104^, ^105^, ^106^, ^107^, ^108^, ^109^, ^110^, ^111^ At a fundamental level, differences between the net charge of the apoE ε2, apoE ε3, and apoE ε4 isoforms confer differences in the isoelectric point (IEP) (i.e., the point at which the surface charge is neutral), which leads to differences in how the isoforms are processed and trafficked by the endolysosomal system.^111^

The match between the IEP of apoE ε4 and the pH of early endosomes suggests that apoE ε4 may lose solubility in the early endosome leading to increased self‐interaction of apoE particles. This, in turn, would be predicted to increase their apparent affinity to clustered apoE receptors within the endosome, hindering the dissociation of the particles from their receptors and thereby impairing normal endosome maturation, recycling, retrograde sorting, and lysosomal degradation. In support of this idea, it has been shown that biochemical and genetic inhibition of a proton leak channel in the early endosome, the Na+/H+ exchanger 6 (NHE6), restores normal apoE and glutamate receptor trafficking through the endosomal compartment.^111^ Furthermore, NHE6 disruption equalizes the phenotypes between apoE isoforms and prevents Aβ plaque accumulation in apoE ε4 KI mice. Targeting endolysosomal dysfunction thus presents a pharmacologically tractable mechanism that may be fundamental to AD risk. Furthermore, taken together these findings suggest that balancing endolysosomal pH homeostasis may be a possible pathway for AD prevention, not only for the increased risk imposed by apoE ε4 but also for other forms of AD.^109^

### 8.3 Targeting microglial pathways downstream of apoE/TREM2

The Target Enablement to Accelerate Therapy Development for AD (TREAT‐AD) initiative has developed a strategy for small‐molecule targeting of pathways downstream of apoE and TREM2. Analysis of apoE/TREM2 signaling networks identified inositol polyphosphate‐5‐phosphatase (INPP5D), also known as SHIP1, to have a significant genetic association with LOAD. INPP5D/SHIP1 is co‐expressed with 73 other genes in an AD immune response module, selectively expressed in microglia, and postulated to limit TREM2‐mediated microglia activation. Furthermore, studies using the 5xFAD mouse model show that INPP5D/SHIP1 is upregulated as Aβ pathology progresses and that INPP5D/SHIP1 haploinsufficiency normalizes AD‐related neuropathology and behavioral deficits, which suggests that inhibition of INPP5D/SHIP1 early in AD would increase TREM2 signaling and microglial protective functions, resulting in reduced rate of disease progression and cognitive decline in AD.^112^

A high‐throughput small‐molecule screen (HTS) for INPP5D/SHIP1 inhibitors using co‐purified INPP5D/SHIP1 identified a lead candidate chemical probe, called compound 23. In vitro studies in primary mouse microglia demonstrated concentration‐dependent effects of compound 23 on INPP5D/SHIP1 signaling, a lack of toxicity, and good exposure in brain and plasma. Next steps include additional structure‐activity studies and development of a lead clinical compound. Studies are also underway to identify inhibitors of additional neuroinflammation targets and, in parallel, to develop relevant biomarkers for INPP5D/SHIP1 and other identified targets.^113^ In addition, a “Target Enablement Package,” available on the AD Knowledge Portal has been recently released that includes data, methods, and research tools to catalyze further drug discovery efforts by academic and industry researchers.^114^

### 8.4 Novel interventions targeting apoE‐heparan sulfate proteoglycan interactions

Compared to apoE ε3, apoE ε4 displays a higher affinity to heparan sulfate proteoglycans (HSPGs), whereas apoE ε2 and apoE3‐Ch display lower binding affinities when used heparin‐affinity chromatography to model this interaction.^20^ This observation suggests that the protective effect of the apoE3‐Ch variant in the Colombian PSEN1 FAD kindred may be mediated by diminished interactions between apoE‐Ch and HSPGs. Marino and colleagues generated anti‐apoE‐HSPG antibodies to mimic the reduced apoE‐Ch‐HSPG interaction, and characterized them using both biomolecular approaches. Among the antibodies screened for inhibition of binding, the top candidate, 7C11, was tested in vitro and in vivo. Notably, 7C11 reduced the cytotoxicity of apoE4 in vitro and rescued apoE4‐induced tau pathology in vivo. Recently, a study using isogenic hiPSC‐derived neurons with apoE4 or apoE4‐Ch showed that apoE4‐Ch reduced HSPG‐mediated tau uptake by neurons in culture.^115^ Together, these findings suggest that developing novel inhibitors of apoE‐HSPGs interactions might lead to effective disease‐modifying therapies for AD.^116^

### 8.5 APOE‐targeted epigenome therapy

A global reduction in overall brain *APOE* levels may have beneficial effects on AD pathogenesis.^99^, ^117^, ^118^, ^119^, ^120^ Consistently, integrative single nucleus multiomic analysis revealed an overexpression of APOE ε3 in specific cellular subtypes in AD brains versus control, as well as more open chromatin in several genomic sites linked to the promoter of the *APOE* gene, which implies disease‐associated cis‐regulatory elements.^121^ In addition, a new study suggested that apoE ε4 drives AD risk through a gain of abnormal function (contrary to the earlier mentioned loss), providing further support that reducing apoE ε4 levels is a promising therapeutic strategy.^97^ These findings informed the development of an epigenome therapeutic approach for treating AD based on a modification of the CRISPR/Cas9 technology and delivered by an all‐in‐one viral vector platform that specifically recognizes the *APOE* ε4 allele and represses and fine‐tunes *APOE* ε4 expression. Homozygous *APOE* ε4 and *APOE* ε3 hiPSC‐derived cholinergic neurons and organoids showed that expression of the *APOE* ε4 allele, but not the *APOE* ε3 allele, was specifically repressed. In vivo studies following injection into mouse hippocampus found that apoE protein expression was reduced by approximately 70% and that the repression effect exhibited sex‐specific differences. This novel epigenome therapy platform offers the opportunity for refinement to develop gene‐, allele‐, cell type‐, and population‐specific therapies, thereby advancing strategies for precision medicine in LOAD.^122^

### 8.6 Phase 1 AAV gene therapy in patients with APOE ε4 homozygote AD

While *APOE* ε4 is associated with great AD risk and earlier age of onset, emerging evidence suggests that *APOE* ε2 has protective effects against AD. Lexeo Therapeutics has a clinical program that is investigating their AAVrh10 gene therapy candidate, LX1001, in patients with homozygous *APOE* ε4‐associated AD. LX1001 is designed to deliver into the CSF and express the protective APOE ε2 gene. Administering the *APOE* ε2 gene to *APOE* ε4 homozygous individuals has the potential to address several pathways that are involved in the progression of AD disease.^101^, ^123^

LX1001 is being evaluated in an open‐label, dose‐escalation Phase 1/2 clinical trial in *APOE* ε4 homozygous individuals who are 50 years of age or older and have mild cognitive impairment (MCI) to moderate dementia and biomarkers consistent with AD. The primary endpoint is safety; additional measures include expression of *APOE* ε2 in the brain, CSF biomarkers (Aβ42, t‐tau, p‐tau), Aβ and tau PET scans, quantitative MRI, and cognitive testing. In the first dose cohort in the trial, the study investigators observed a consistent trend toward improvement in AD CSF biomarkers, such as total tau and phosphorylated tau. Investigators have also observed expression of the protective apoE ε2protein in all patients in the first dose cohort with follow‐up data. Among all patients, treatment with LX1001 has been well‐tolerated with no serious related adverse events reported as of July 2023. Although these initial data are promising, evaluation of the dose‐response relationship, effects of higher doses, and inclusion of more patients will provide additional insight into this therapeutic approach and its potential clinical impact (Clinical Trial NCT03634007).

#### 8.6.1 Hormone replacement therapy in women with APOE ε4

Estrogen decline during the menopausal transition is emerging as a key factor enhancing AD risk in women.^124^ Estrogen regulates multiple neurophysiological processes, including cerebrovascular function, BBB integrity, synaptic plasticity, neuroinflammation, and brain energy metabolism. Furthermore, estrogen and progesterone receptors are expressed in multiple brain regions relevant to cognitive function and AD.^125^ Hormone replacement therapy (HRT) has thus been of interest as a strategy to reduce cognitive decline in women, although clinical trial results have been inconsistent thus far.^126^, ^127^, ^128^ A recently published study by Saleh and colleagues investigated whether HRT would have greater cognitive benefits in *APOE* ε4 compared to non‐*APOE* ε4 women, particularly when introduced early during the menopausal transition. The study found that *APOE* ε4 women on HRT compared to non‐HRT scored higher on delayed memory tests and had larger entorhinal and amygdala volumes. Interestingly, earlier HRT initiation was associated with larger hippocampal volume. Findings thus far emphasize the importance of personalized medicine in AD prevention.^129^

## 9 CONCLUSION

The neurodegenerative disease and biomedical research communities have made significant progress in understanding the structure, function, associated pathologies, and clinical impact of apoE. The “AAIC advancements: APOE conference,” which assembled over 850 participants from 54 countries, helped to facilitate discussions on emerging APOE research, formed collaborative efforts, and provided a forward perspective on the field. The conversations clarified areas in which further study is necessary and worthwhile, including studies on the mechanisms underlying sex‐dependent impacts of apoE isoforms, development of improved animal and cell models of AD and other apoE‐related diseases, clinical impact of modified apoE proteins (e.g., lipidated), and assessment of therapeutics targeting apoE‐related physiological aberrations.

Another major priority for the entire research community is to diversify the patient populations within studies and clinical trials to be representative of the population impacted by APOE‐related conditions, particularly AD. To better understand the neural‐genetic‐environmental interplay underlying apoE‐pathology and improve the livelihood of all individuals, research studies must prioritize understanding the intricacies of APOE in various geographical, racial, and ethnic groups that are impacted. These approaches can improve the many ongoing and future APOE‐focused research studies and therapeutic trials and enable new discoveries that ultimately help prevent, diagnose, and treat‐AD.

## CONFLICT OF INTEREST STATEMENT

C. M. Kloske, M. C. Carrillo, S. Mahinrad, and C. E. Sexton are all full time employees of the Alzheimer's Association. M. Belloy has nothing to disclose. E. E. Blue was was funded by the National Institutes of Health/National Institute on Aging grant number R01AG059737 and is related to her work funded by grant number U01AG058589. Dr. Blue is a member of the Board of Directors for the International Genetic Epidemiology Society. G. R. Bowman holds equity in Decrypt Biomedicine. X. Chen has nothing to disclose. O. Chiba‐Falek is a co‐inventor of the related IP. Duke University filed patent applications for the technology developed in this study. CLAIRIgene has an exclusive, worldwide option agreement from Duke for the related patent portfolio for all fields of use. Dr. Chiba‐Falek is a Co‐Founder at CLAIRIgene, LLC. A. Davis has nothing to disclose. G. Di Paolo is a full‐time employee and shareholder of Denali Therapeutics Inc. F. Garretti has nothing to disclose. D. Gate has nothing to disclose. L. M. Golden has nothing to disclose. J. Heinecke has nothing to disclose. J.Herz is a cofounder of Reelin Therapeutics. Y. Huang has nothing to disclose. C. Iadecola has nothing to disclose. L. A. Johnson has nothing to disclose. T. Kanekiyo has nothing to disclose. C. Karch has nothing to disclose. A.Khvorova is a Co‐Founder of Atalanta. S. Koppes‐den Hertog has nothing to disclose. B. Lamb Co‐Founded of Monument Biosciences. P. E. Lawler has nothing to disclose. Y. Le Guen has nothing to disclose. A. Litvinchuk has nothing to disclose. C. Liu has nothing to disclose. E. Marcora has nothing to disclose. C. Marino has nothing to disclose. D. M. Michaelson has nothing to disclose. J. J. Miller has nothing to disclose. J. M. Morganti has nothing to disclose. P. S. Narayan has nothing to disclose. M. S. Naslavsky has nothing to disclose. M. Oosthoek has nothing to disclose. K. V. Ramachandran has nothing to disclose. A Ramakrishnan has nothing to disclose. A. Raulin has nothing to disclose. A. Robert has nothing to disclose. R. N. M. Saleh has nothing to disclose. N. Shah is an employee of Lexeo Therapeutics. F. Shue has nothing to disclose. I. J. Sible has nothing to disclose. A. Soranno has nothing to disclose. M. R. Strickland has nothing to disclose. J. TCW has nothing to disclose. M. Thierry has nothing to disclose. L. Tsai has nothing to disclose. R. A. Tuckey has nothing to disclose. J. D. Ulrich has nothing to disclose. R. van der Kant has nothing to disclose. N. Wang has nothing to disclose. C. L. Wellington has nothing to disclose. S. C. Weninger has nothing to disclose. H. N. Yassine has nothing to disclose. N. Zhao has nothing to disclose. G. Bu is the Editor‐in‐Chief of Molecular Neurodegeneration and a consultant for SciNeuro Pharmaceutical. A. M. Goate serves on the scientific advisory boards for Genentech and Muna Therapeutics. D. M. Holtzman is an inventor on a patent licensed by Washington University to NextCure on the therapeutic use of anti‐APOE antibodies. Pub. No. US 2022/0411485 A1 Pub date Dec 29, 2022. Title of patent: ANTI‐APOE ANTIBODIES. D.M.H. co‐founded and is on the scientific advisory board of C2N Diagnostics. D.M.H. is on the scientific advisory board of Denali, Genentech, and Cajal Neuroscience and consults for Asteroid. Author disclosures are available in the supporting information.

## Supporting information

Supporting Information
